# High-Grade Prostate Cancer: Favorable Results in the Modern Era Regardless of Initial Treatment

**DOI:** 10.5402/2012/596029

**Published:** 2012-01-31

**Authors:** Emma H. Ramahi, Gregory P. Swanson, Matthew W. Jackson, Fei Du, Joseph W. Basler

**Affiliations:** ^1^The University of Texas Health Science Center San Antonio, 7703 Floyd Curl Drive MC7889, San Antonio, TX 78229, USA; ^2^Department of Radiation Oncology, Radiology, and Urology, The University of Texas Health Science Center San Antonio, 7703 Floyd Curl Drive MC7889, San Antonio, TX 78229, USA; ^3^Department of Epidemiology and Biostatistics, The University of Texas Health Science Center San Antonio, 7703 Floyd Curl Drive MC7889, San Antonio, TX 78229, USA; ^4^Department of Urology, The University of Texas Health Science Center San Antonio, 7703 Floyd Curl Drive MC7889, San Antonio, TX 78229, USA

## Abstract

*Purpose*. We performed a retrospective study to determine the outcome of a modern cohort of patients with high-grade (Gleason score ≥ 8) prostate cancer treated with radical prostatectomy, radiation therapy, or hormone therapy. *Methods*. We identified 404 patients in the South Texas Veteran's Healthcare System Tumor Registry diagnosed with high grade prostate cancer between 1998 and 2008. Mean follow-up was 4.62 ± 2.61 years. End points were biochemical failure-free survival, overall survival, metastasis-free survival, and cancer-specific survival. *Results*. 5-year overall survival for patients undergoing radical prostatectomy, radiation therapy, and hormone therapy was 88.9%, 76.3%, and 58.9%, respectively. 5-year metastasis-free survival for patients undergoing radical prostatectomy, radiation therapy, and hormone therapy was 96.8%, 96.6%, and 88.4%, respectively, and 5-year cancer-specific survival was 97.2%, 100%, and 89.9%, respectively. Patients with a Gleason score of 10 and pretreatment prostate-specific antigen > 20 ng/mL had decreased 5-year biochemical failure-free and cancer-specific survival. Patients with a pretreatment prostate-specific antigen > 20 ng/mL had decreased 5-year overall survival. *Discussion*. Even for patients with high-grade disease, the outcome is not as dire in the modern era regardless of primary treatment modality chosen. While there is room for improvement, we should not have a nihilistic impression of how these patients will respond to treatment.

## 1. Introduction

In the modern era of prostate specific antigen (PSA) screening, thousands of men are diagnosed with prostate cancer each year that would have previously escaped detection. Of the estimated 218,000 American men diagnosed with prostate cancer in 2010 [1], men with high-grade disease (Gleason 8–10) are at greatest risk for adverse outcomes. Gleason score has long been shown to correlate well with risk for local extension, metastasis, and death [2]. While clinicians generally agree that men with high-grade disease require prompt therapy to limit disease-specific morbidity and mortality, the best treatment for these men remains controversial. As a result, they are treated with a variety of different options.

Radical prostatectomy (RP) and radiation therapy (RT) remain the standard modalities for treatment of patients with prostate cancer. Hormone therapy (HT) is used either in combination with more definitive therapy or considered palliative treatment when used alone. Regardless of treatment, patients with high-grade prostate cancer have historically experienced poor outcomes. Prior studies have shown poor survivorship rates for those treated with either RP [3] or RT alone [4]. Patients who are surgical candidates for radical prostatectomy are often younger and lack the comorbidities of their counterparts receiving RT. These and other confounders limit retrospective studies comparing efficacies of RP and RT. One study showed no significant difference in rates of PSA failure at 10 years in high-risk patients treated with RP or RT [5]. Large prospective, randomized studies are required to definitively compare these two standards of treatment, but due to a variety of obstacles, they remain notably absent from the current literature.

Any discussion on treatment modalities for high-grade prostate cancer would be incomplete without consideration of HT. Though traditionally considered a palliative treatment, a small number of studies have suggested primary HT affects meager improvements in either overall survival [6] or prostate cancer-specific survival [7]. Further investigation of HT is warranted to elucidate its role as singular therapy and its contribution to combined therapy with RP and RT.

There are few currently published studies examining treatment outcomes for an entire cohort of patients with high-grade disease from a single institution. Thus, in this retrospective analysis of patients diagnosed with Gleason 8–10 prostate cancer in the South Texas Veteran's Health Care System, we aim to examine the effects of pretreatment disease characteristics and initial treatment modality on biochemical failure-free survival (BFFS), metastasis-free survival (MFS), cancer-specific survival (CSS), and overall survival (OS) in the modern era. It would be unrealistic in this retrospective review to directly compare outcomes between patients receiving RP, RT, and HT. Most studies report these cohorts separately, but we think that it is informative to look at the outcome for the entire high-risk population across the various treatment modalities. We hypothesize that high-grade patients from this modern cohort will have less failure and better survival than those previously reported.

## 2. Methods

After receiving approval from the institutional review board, we reviewed all patients in the South Texas Veteran's Healthcare System Tumor Registry diagnosed with prostate cancer between January 1, 1998, and December 31, 2008, capturing 404 patients with Gleason score ≥ 8 on biopsy or on radical prostatectomy pathology. Sixty-one surgical patients had a biopsy Gleason score < 8 but were found to have Gleason 8–10 disease on RP pathology. Statistical analysis was performed both with and without the inclusion of these patients, and their inclusion did not lead to significant differences in outcome measure comparison.

Patient information recorded from the medical record included age at diagnosis, race, PSA immediately prior to diagnosis, information from the original biopsy report such as Gleason score as determined by the original attending pathologist, percentage of positive cores, and whether positive cores came from one or both sides of the prostate. The primary treatment of prostate cancer was recorded as RP, RT, or HT. Furthermore, surgical pathology characteristics, as interpreted and recorded by the original attending pathologist, were recorded including presence of positive lymph nodes, positive seminal vesicles, extracapsular extension, and positive surgical margins. Finally, the use of any adjuvant or neoadjuvant treatment including HT, RT, or chemotherapy was recorded. Patients were excluded from analysis if they had metastatic disease at the time of diagnosis as identified by bone scan, computed tomography scan, or magnetic resonance imaging. Patients were also excluded from analysis if they did not receive treatment for their prostate cancer or if they were lost to follow up before posttreatment PSA values could be obtained.

Biochemical failure was defined differently depending on the primary treatment. For surgical patients, biochemical failure was defined by PSA ≥ 0.2 ng/mL followed by a confirmatory PSA value ≥ 0.2 ng/mL [8]. For radiation patients, biochemical failure was defined by a PSA rise 2.0 ng/mL above the nadir [9]. Patients receiving RP or RT were also considered to have failed on the date salvage treatment was initiated. For patients receiving HT, failure was defined by the development of metastasis or the initiation of another treatment modality. Patients who had only received a luteinizing hormone releasing hormone (LHRH) agonist were not recorded as failing with a rising PSA until either their PSA continued to rise after additionally having received an antiandrogen, or they failed another way. The choice of initial treatment was determined by the treating physician after receiving informed consent from the patient. Reasons a patient may receive HT as opposed to one of the localized treatment modalities, RT or RP, include, but are not limited to, poor candidacy for surgery or radiation due to medical comorbidities, bulk of disease, or patient preference.

### 2.1. Statistical Methods

Continuously distributed data were summarized with the sample size, mean, and standard deviation (SD), and categorical data were described with counts and percentages. Data were grouped by primary treatment modality, RP, RT, or HT. Groups were contrasted on continuously distributed outcomes with Kruskal-Wallis tests. The significance of associations between categorical outcomes and primary treatment was assessed with Pearson's chi-square test. Treatment categories were contrasted with regard to biochemical failure-free, metastasis-free, cancer-specific, and overall survival using a logistic regression model adjusted for age, Gleason score, pretreatment PSA, and positive cores. All statistical testing was 2-sided with a significance level of 5%. SAS Version 9.2 for Windows (SAS Institute, Cary, North Carolina) was used throughout.

## 3. Results

Included for analysis were 404 patients with high-grade prostate cancer and a mean follow-up time of 4.62 ± 2.61 years. Not unexpectedly, the patient characteristics for the various treatment modalities varied (Table 1). Patients receiving RP were significantly younger than those receiving RT or HT (64.6 ± 7.2 years compared with 70.9 ± 8.4 and 77 ± 8.1, resp.; *P* < 0.001). Patients in the treatment groups did not significantly differ in terms of race or Gleason score, but surgical patients had a lower mean pretreatment PSA than those receiving RT or HT (10.3 ± 11.9 ng/mL compared with 15.5 ± 27.1 and 48.2 ± 79.4, resp.; *P* < 0.001). Patients receiving HT also had a greater percentage of positive biopsy cores than those receiving RP or RT (60% positive compared with 50% and 40%, resp.; *P* < 0.001) (Table 1). Overall, patients receiving androgen ablation were older and had bulkier cancers than those undergoing radiation therapy or surgery.

**Figure 1:**
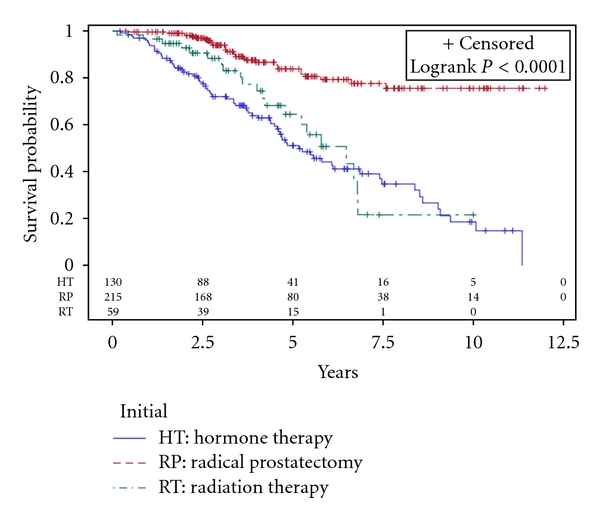
Overall survival by initial treatment category.

Gleason score and pretreatment PSA were associated with significant differences in outcomes (Table 2). Patients with increasing Gleason score had decreasing 5-year BFFS and CSS, and patients with increasing pretreatment PSA had decreasing 5-year OS, BFFS, and CSS. Patients undergoing surgery had a significant better overall survival, even after adjusting for age, Gleason score, pretreatment PSA, and percentage of positive cores. For the entire cohort, the 5-year overall survival, metastasis-free survival, and cause-specific survival were 77.5%, 94.1%, and 95.3%, respectively (Table 3). Importantly, the metastasis-free and cause-specific survival at 5 years were favorable across all the treatment modalities. Overall survival probabilities are shown via a Kaplan-Meier curve in Figure 1.

## 4. Discussion

Most currently published studies report long-term failure and survival concerning patients diagnosed from the 1970s to early 1990s. Though the extended follow-up afforded to these earlier studies allows for better characterization of the long natural history of prostate cancer, advances in surgical, radiation, and hormone therapy have led some to wonder whether previously published survival estimates are applicable to modern high-risk patients. Ours is one of the largest modern cohorts including patients with high-grade prostate cancer treated with RP, RT, or HT at a single institution.

Overall, the patients in the entire cohort did surprisingly well with a low rate of metastatic disease and cancer death. Specifically, the 5-year rate of metastatic disease and cancer death was <10%. There are really no comparative studies like ours that look at the experience across the different treatment modalities, but given the differences in patient selection, the patients have done very similarly. It is interesting to further evaluate each of the three treatment cohorts.

### 4.1. RP for High-Grade Prostate Cancer

For surgical patients in our series, 5-year CSS was 97.2%. This is significantly more optimistic than previous high-grade prostate cancer surgical series reporting 10-year CSS ranging from 67 to 85% [3, 10–13].

Lau et al. from the Mayo Clinic reported BFFS, OS, and CSS of 36%, 67%, and 85%, respectively, in a cohort of 407 patients with pathologic Gleason score ≥ 8 who underwent RP from 1987 to 1996 [3]. In addition to the time period of diagnosis, differences exist between these patients and those in our series. Patients in Lau's series had a higher percentage of lymph node positive disease (27% versus 4.6% in our series). Likely due to the advanced pathologic stage, 45% of patients in Lau's series received adjuvant treatment, whereas only 10.2% of patients in our series received adjuvant, concurrent, or neoadjuvant RT, HT, or chemotherapy.

More recently, Tewari et al. published long-term survival outcomes on a cohort of 453 patients ≤ 75 years old with high-grade prostate cancer treated with RP, RT, HT, or active surveillance between 1980 and 1997, 119 of whom underwent RP [14]. Using propensity risk analysis, Tewari et al. reported a risk of cancer-specific death for surgical patients that was 49% lower than patients receiving RT and sixty-eight percent lower than patients receiving HT or active surveillance (*P* = 0.53 and < 0.001, resp.). Though unadjusted survival analysis suggested that patients undergoing RP and RT had better CSS than those receiving HT, after adjusting the logistic regression model for age, Gleason score, and pretreatment PSA, our data showed no significant difference in 5-year CSS for patients undergoing RP, RT, and HT (97.2%, 100%, and 89.9%, resp.; *P* = 0.91) (Table 3). Overall, our results support that patients with high-risk prostate cancer can do well with surgery as the primary approach.

### 4.2. RT for High-Grade Prostate Cancer

In our series, 5-year CSS for RT patients was 100%. Roach et al. report a dismal 44% 10-year CSS in patients treated with RT alone pooled from four prospective phase III randomized RTOG trials between 1975 and 1992 [4]. In contrast, 50.8% of the RT patients in our series received either adjuvant, concurrent, or neoadjuvant HT. 68% of patients from the RTOG trials received a total dose less than 70 Gray (Gy), and as most patients were diagnosed before the PSA era, most presented with T3 disease, and 36% of those with T1-T2 disease had positive lymph nodes [4]. In our series, 66.1% received external beam radiation therapy (EBRT) with a mean dose of 75.5 Gy (range 68.4 to 77 Gy), 5.1% received brachytherapy, 18.6% received EBRT + brachytherapy boost, and 10.2% had incomplete records.

Kupelian et al. reported equivalent 5-year BFFS when comparing RP, EBRT, brachytherapy, or combination of EBRT and brachytherapy as long as the EBRT dose met or exceeded 72 Gy [15]. D'Amico et al. likewise found no significant difference in BFFS between RP and EBRT after 8 years [5]. Beyer and Brachman reported improved 5-year BFFS with EBRT compared to brachytherapy for high-grade patients [16], but as only 3 patients in our series received brachytherapy alone, we could not observe any difference.

Stock et al. recently published a contemporary case series of 181 patients with Gleason scores 8–10 treated with ^103^Pd implant, 45 Gy EBRT, and 9 months of HT between 1994 and 2006. With reported 8-year BFFS, OS, MFS, and CSS of 73%, 79%, 80%, and 87%, respectively, Stock et al. show similarly optimistic outcomes for high-grade prostate cancer patients treated with RT in the modern era [17].

Overall, our results support that patients with high-risk prostate cancer can do well with radiation therapy as the primary approach.

### 4.3. HT for High-Grade Prostate Cancer

There is a dearth of publications in the current literature describing failure and survival of patients treated primarily with HT, and the few studies that do exist report OS of 13%–48% [6, 7, 18]. Lu-Yao et al. failed to show significantly improved 10-year OS when compared with conservative management 17.3% versus 15.3%, respectively, in patients with poorly differentiated disease [7]. However, Studer et al. reported improved OS, though no effect on CSS or symptom onset, when androgen ablation was initiated immediately rather than being deferred in an EORTC trial enrolling patients not suitable for definitive treatment [6].

Our data show a more favorable outcome for patients treated with HT. This may be partly due to the fact that of the 36 patients failing HT, 18 went on to receive additional treatment (11 received RT, 6 received chemotherapy, 1 received salvage RP). Also, in some cases, patients failing an LHRH agonist were simply observed with a rising PSA and died before metastatic disease became manifest. To that end, many of these patients were treated with androgen ablation due to advanced age and comorbidities along with high PSA worrisome for undetected metastasis. With a median survival of only 3.7 years, many died of intercurrent disease.

Our results support that in elderly patients with a projected short longevity, primary androgen ablation can provide reasonable control even in patients with bulky high-grade disease.

Overall, the principle strength of this study is the large number of prostate cancer patients from a single institution in the modern diagnostic and treatment era. Study limitations include those inherent to any retrospective analysis. Selection of treatment was at the discretion of the original treating physician and factors such as age, comorbidities, prostate size, and patient preference affected candidacy for surgery, radiation, or hormone therapy. Selection bias and noncongruent definitions of biochemical failure prevent effective comparison between RP, RT, and HT treatment groups. Additionally, a mean follow-up of 4.6 ± 2.7 years is not long enough to capture all deaths from prostate cancer or development of metastatic disease. Though all our patients were diagnosed and followed within the Veterans Health Care System, a central pathologic review was not performed, and Gleason scores as well as other pathologic features were recorded as interpreted by the original attending pathologists. Still, even with these limitations, our patients have done fairly well with what would be universally considered significant disease.

## 5. Conclusions

Historically, patients with a Gleason score of 8–10 have done very poorly. However, in the modern era, regardless of the initial treatment, the outcome is not as dire. Possible reasons are less bulky tumors at diagnosis due to the advent of routine PSA screening and increased awareness of prostate cancer in the community, as well as improved surgical and radiation techniques. Prospective, randomized phase III studies comparing all primary treatment groups would provide the best information with which we counsel our high-grade patients about any differences in the various treatment modalities. In the absence of such trials, this retrospective review can help provide insight into what patients could possibly expect from RP, RT, and HT in the modern treatment era. While there is room for improvement, we should not have a nihilistic impression of how these patients will respond to treatment.

## Figures and Tables

**Table 1:** Comparison of patient demographics and pretreatment features.

	Surgery	Radiation	Hormone therapy	Total	*P* value
Age					<0.001^1^
*N*	216	59	129	404	
Mean (SD)	64.6 (7.2)	70.9 (8.4)	77 (8.1)	69.5 (9.5)	
Race, *N* (%)					0.48^2^
White	161 (74.5)	40 (67.8)	92 (71.3)	293 (72.5)	
Black	14 (6.5)	7 (11.9)	16 (12.4)	37 (9.2)	
Other	3 (1.4)	0 (0)	1 (0.8)	4 (1)	
Unknown	38 (17.6)	12 (20.3)	20 (15.5)	70 (17.3)	
Total	216	59	129	404	
Gleason score, *N* (%)					0.37^2^
8	84 (54.2)	33 (55.9)	63 (48.8)	180 (52.5)	
9	67 (43.2)	22 (37.3)	57 (44.2)	146 (42.6)	
10	4 (2.6)	4 (6.8)	9 (7)	17 (5)	
Total^3^	155	59	129	343	
PSA at diagnosis (ng/mL)					<0.001^1^
*N*	208	57	126	391	
Mean (SD)	10.3 (11.9)	15.5 (27.1)	48.2 (79.4)	23.3 (50)	
Fraction of positive cores					<0.001^1^
*N*	214	59	126	399	
Mean (SD)	50% (30%)	40% (30%)	60% (30%)	50% (30%)	

^1^Kruskal-Wallis Test.

^2^Pearson's Chi-Square Test.

^3^61 surgical patients had a Gleason score of <8 on biopsy but ≥8 on radical prostatectomy pathology and thus were included for analysis.

**Table 2:** Comparison of outcome measures by pretreatment features and primary treatment.

	Outcome measures					
	5-year OS	*P* value^1^	5-year BFFS	*P* value^1^	5-year CSS	*P* value^1^
Gleason score						
8	77.2%	0.71	75.6%	<0.001	96.1%	0.001
9	74%		58.2%		95.9%	
10	70.6%		41.2%		76.5%	
Pretreatment PSA (ng/mL)						
<10	80.7%	0.002	72.1%	0.001	98%	<0.001
10–20	83.3%		61.1%		100%	
>20	63.6%		51.1%		84.1%	
Extent of disease at diagnosis						
Unilateral	79.7%	0.53	67.8%	0.54	97.2%	0.28
Bilateral	77%		64.9%		95%	
Percentage of positive cores						
<50%	79.8%	0.30	71.3%	0.02	95.7%	0.69
≥50%	75.5%		60.6%		94.9%	
Months from diagnosis to treatment						
<3	76.5%	0.08	62.6%	0.29	94.2%	0.43
3–12	81.6%		70.2%		97.2%	
>12	60%		70%		95%	

^1^OS: Overall Survival; BFFS: Biochemical Failure-Free Survival; MFS: Metastasis-Free Survival; CSS: Cancer-Specific Survival.

^2^Pearson's Chi-Square test.

**Table 3:** 5-year outcome measures by primary treatment.

	Surgery	Radiation	Hormone therapy	Total	*P* ^1^
5-year OS, %	88.9%	76.3%	58.9%	77.5%	0.01
5-year BFFS, %	54.2%	81.4%	77.5%	65.6%	<0.001
5-year MFS, %	96.8%	96.6%	88.4%	94.1%	0.24
5-year CSS, %	97.2%	100%	89.9%	95.3%	0.8

^1^OS: Overall Survival; BFFS: Biochemical Failure-Free Survival; MFS: Metastasis-Free Survival; CSS: Cancer-Specific Survival.

^2^Based on a logistic regression model adjusted for age, Gleason score, pretreatment PSA, and positive cores.
